# Detection of Extended-Spectrum β-Lactamases among *Acinetobacter Baumannii* Isolated from Hospitals of Qazvin, Iran

**DOI:** 10.4314/ejhs.v31i2.4

**Published:** 2021-03

**Authors:** Mina Zarabadi-Pour, Amir Peymani, Narges Habibollah-Pourzereshki, Mohammad Reza Sarookhani, Ali Akbar Karami, Amir Javadi

**Affiliations:** 1 Student Research Committee, Qazvin University of Medical Sciences, Qazvin, Iran; 2 Medical Microbiology Research Center, Qazvin University of Medical Sciences, Qazvin, Iran; 3 Cellular and Molecular Research Center, Qazvin University of Medical Sciences, Qazvin, Iran; 4 Department of Urology, School of Medicine, Qazvin University of Medical Sciences, Qazvin, Iran

**Keywords:** Acinetobacter baumannii, ESBL, repetitive extragenic palindromic-PCR

## Abstract

**Background:**

Acinetobacter baumannii is a major contributor to nosocomial infections. Extended-spectrum β-lactamase (ESBL)-producing A. baumannii is spreading worldwide. We aimed to determine the frequency of ESBL-encoding genes in clinical isolates of A. baumannii and to access their clonal relationship by repetitive extragenic palindromic-PCR (rep-PCR).

**Methods:**

In this descriptive cross-sectional study, 203 isolates of A. baumannii were collected from Qazvin hospitals. The Identification of isolates was performed by standard laboratory methods. To verify ESBL production, all isolates were screened by disk agar diffusion and confirmed by the combined disk method. Subsequently, ESBL-encoding genes were detected by PCR and sequencing. Possible clonal association of ESBL-producing isolates was evaluated using rep-PCR.

**Results:**

Two hundred (98.5%) isolates showed reduced susceptibility to one of the antibiotics used in the ESBL screening test, of which 127 isolates (62.6%) produced ESBL. PCR results showed bla_OXA-1_ (20.5%) was the most prevalent gene followed by bla_TEM-1_ (20%), bla_GES-1_ (15.7%), bla_CTX-M-15_ (7.9%), and bla_PER-1_ (1.6%). Rep-PCR results revealed that ESBL-producing isolates belonged to clones A (85%), B (13.4%), and C (1.6%).

**Conclusion:**

Our study showed the significant presence of bla_OXA-1_, bla_TEM-1_, bla_GES-1_, bla_CTX-M-15_, and bla_PER-1_ genes in ESBL-producing A. baumannii isolates in the studied hospitals. This is the first report on the emergence of bla_OXA-1_ gene in these isolates in Iran. The use of comprehensive antimicrobial treatment guidelines based on laboratory data and appropriate infection control interventions are essential.

## Introduction

*Acinetobacter baumannii* (*A. baumannii*), an opportunistic pathogen involved in health care-associated infections, is a gram-negative, non-fermentative, aerobic bacterium. These organisms are major causes of infections such as urinary tract infection (UTI), bacteremia, meningitis, pneumonia, and burn infections (1,2). In the last decade, *Acinetobacter* strains have become resistant to most antibiotics, especially in Asia (3–5). Over use and/or misuse of antimicrobials is leading to increasing the emergence of multidrug resistant strains of *A. baumannii* worldwide. This organism has become resistant to several antibiotic classes including β-lactam, aminoglycoside, and quinolones (6). Due to its high ability to develop resistance and create MDR strains, the treatment of these infections has become difficult and costly and sometimes impossible (7). β-lactam antibiotics have been frequently used for treating *A. baumannii* infections (8). Resistance to β-lactams in *A. baumannii* isolates is due to intrinsic and acquired mechanisms including enzymatic alteration, mutation in target genes, alteration of outer membrane permeability and increased efflux pump activity (9). The most important mechanism of β-lactam resistance in Gram-negative bacteria is the production of β-lactamase enzymes that has posed many challenges in the antibiotic treatment of infections caused by these organisms (10). The Ambler classification divides the β-lactamases into four major molecular classes (A-D). The extended-spectrum β-lactamases (ESBLs) belong to class A and can hydrolyze first, second and third-generation of cephalosporins, penicillins, and monobactams but are inhibited by clavulanate (11). The important ESBL families in many enteric gram-negative bacilli are CTX, TEM, OXA, SHV, GES, VEB and PER (12). The ESBL-encoding genes are often found on plasmids that allow the spread of resistance factors between these isolates and other organisms that cause healthcare-associated infections (13).

Currently, given the high prevalence and rapid worldwide spread of multiple drug resistant (MDR) *A. baumannii* isolates, mortality in hospitalized patients, especially in the intensive care units (ICUs), has increased significantly (14). Several recent studies suggest that the release of ESBL-encoding genes could complicate the treatment of *A. baumannii* infections because of limited therapeutic choices (15). Therefore, we aimed to determine the prevalence of ESBLs and the genes encoding these enzymes including *bla*_GES_, *bla*_VEB_, *bla*_SHV_, *bla*_TEM_, *bla*_CTX_, *bla*_OXA_, and *bla*_PER_ in clinical isolates of *A. baumannii* as well as to assess their clonal relationship in teaching hospitals of Qazvin, Iran.

## Methods

**Bacterial isolates**: The current study was a descriptive cross-sectional study done during July 2017 to September 2018 in two teaching hospitals in Qazvin, Iran. In total, 203 *A. baumannii* isolates (one isolate per patient) were collected from different clinical specimens including urine, blood, trachea, wounds and from patients admitted to infectious diseases intensive care units, neurosurgery, and surgery wards. Identification of isolates was performed using standard microbiological and biochemical tests including gram staining and microscopic examination, culture on MacConkey agar medium, lactose fermentation, negative oxidase, immobility on SIM medium, alkaline slant/alkaline butt (ALK/ALK) pattern on triple sugar iron (TSI) and no pigment production (16). The identification was further confirmed by detecting of the intrinsic *bla*_OXA-51-like_ and *gltA* (encoding bacterial citrate synthase) genes as previously described (17–18). All isolates were then stored at -70 °C in tryptic soy broth (TSB) medium with 20% glycerol until subsequent tests.

**Ethical considerations**: This study was approved by the Ethics Committee of Qazvin University of Medical Sciences (code IR.QUMS.REC.1396.696). Written informed consent was obtained from all individuals enrolled in the study.

**Detection of ESBL-producing isolates by phenotypic methods**: First, all isolates were prepared using standard Disk Agar Diffusion (DAD) method using cefpodoxime (30 µg), ceftazidime (30 µg), ceftriaxone (30 µg), cefotaxime (30 µg) and aztreonam (30 µg) discs (Mast Diagnostics Group Ltd, Merseyside, UK) according to Clinical and Laboratory Standard Institute (CLSI) guidelines (19). Then, isolates that reduced susceptibility to one of the used antibiotics were screened for confirmation of ESBL production using the combined disc method. In this method, discs containing ceftazidime or cefotaxime (30 µg) with and without clavulanic acid (10 µg) were used. An ≥5 mm increase in zone of inhibition for ceftazidime/clavulanate (30/10 µg) and cefotaxime/clavulanate (30/10 µg) compared to the zone diameter in the absence of clavulanate was considered to be an ESBL producer. For the quality control of susceptibility testing, *A. baumannii* standard isolate American Type Culture Collection (ATCC) 19606 was used.

**Detection of ESBL-encoding genes by PCR and Sequencing**: The amplification of *bla*_OXA_, *bla*_GES-1_, *bla*_VEB-1_, *bla*_SHV_, *bla*_TEM_, *bla*_CTX-M_, and *bla*_PER-1_ genes was performed using specific primers through PCR (Table 1). Total DNAs was first extracted by the extraction kit (Bioneer Company, South Korea) according to the manufacturer's instructions. Then, the PCR was done in a total reaction volume of 20 µl, containing 8 µl of DNA polymerase 2x Master Mix (Ampliqon, Odense, Denmark), 0.5 µM were prepared from each reverse and forward primer, 10 µl distilled water and 1µL DNA template (50 g concentration). PCR amplification of each of the studied genes was done using a thermocycler (Applied Biosystems, USA) under the following program: initial denaturation temperature of 96 °C for 5 min, 30 cycles including denaturation temperature of 95 °C for 1 min, specific annealing temperature for each primer for 1 minutes, extension temperature of 72 °C for 1 min.

**Table 1:** Sequences of primers used in this study

Target genes	Primer sequence (5′-3′)	Annealing temperature (°C)	References
*bla*_SHV_	F: GGTTATGCGTTATATTCGCC R: TTAGCGTTGCTTGTGCTC	50	(20)
*bla*_TEM_	F: ATGAGTATTCAACATTTCCG R: CTGACAGTTACCAATGCTTA	50	(21)
*bla*_VEB-1_	F: CGACTTCCATTTCCCGATGC R: GGACTCTGCAACAAATACGC	55	(22)
*bla*_PER-1_	F: AATTTGGGCTTAGGGCAGAA R: ATGAATGTCATTATAAAAGC	53	(23)
*bla*_GES-1_	F: ATGCGCTTCATTCACGCAC R: CTATTTGTCCGTGCTCAGG	53	(24)
*bla*_OXA-4_	F: TCAACAGATATCTCTACTG TT R: TTTATCCCATTTGAATATGGT	50	(25)
*bla*_OXA-10_ (group I)	F: AGCCGTTAAAATTAAGCCC R: CTTGATTGAAGGGTTGGGCG	56	(22)
*bla*_OXA-2_ (group II)	F: GCCAAAGGCACGATAGTTGT R: GCGTCCGAGTTGACTGCCGG	62	(26)
*bla*_OXA-1_ (group III)	F: AGCCGTTAAAATTAAGCCC R: CTTGATTGAAGGGTTGGGCG	53	(27)
*bla*_CTX-M-1_	F: ATGGTTAAAAAATCACTGCGTC R: TTGGTGACGATTTTAGCCGC	55	(28)
*bla*_CTX-M-2_	F: ATGATGACTCAGAGCATTCG R: TGGGTTACGATTTTCGCCGC	55	(28)
*bla*_CTX-M-8_	F: ACTTCAGCCACACGGATTCA R: CGAGTACGTCACGACGACTT	55	(28)
*bla*_CTX-M-9_	F: ATGGTGACAAAGAGAGTGCA R: CCCTTCGGCGATGATTCTC	55	(28)
rep-PCR	F: IIIGCGCCGICATCAGGC R: ACGTCTTATCAGGCCTAC	45	(29)

A final extension step of 8 minutes at 72ºC was also performed. PCR products were visualized by electrophoresis on agarose gel (1%) using gel documentation system (UVtec, UK). The purified PCR products sent to the Macrogen Company (Seoul, Korea) for sequencing and then the data were analyzed online on The Basic Local Alignment Search Tool (BLAST) available at National Center for Information Biotechnology.

**Rep-PCR analysis**: Rep-PCR was done in a final volume of 20 µl, including 10 µl of distilled water, 8 µl of Master Mix (Ampliqon, Odense, Denmark), 0.5 µl of each forward and reverse primers (Table 1), 1 µl of the template DNA. The conditions of the amplification were as follows: initial denaturation of 95 °C for 3 minutes; then 35 cycles including denaturation temperature of 92 °C for 1 min, specific annealing temperature of 36 °C for 1 min, extension time of 72 °C for 10 min. A final extension step of 16 minutes at 72ºC was also performed. The PCR products were then analyzed by gel electrophoresis using 2% agarose gel and the results were visualized and the isolates with similar patterns (less than two bands difference) were assigned to the same clonal groups (30).

## Results

**Patients and isolates data**: In this study, *A. baumannii* isolates were collected from the trachea (109, 53.8%), urine (33, 16.4%), blood (32, 15.8%) and wound (12, 5.9%), and sputum (17, 4.8%), respectively. The isolates were obtained from 122(60.1%) patients admitted to ICUs: 39(19.2%) to internal medicine, 25(12.3%) to infectious diseases, 11(5.4%) to neurosurgery and 6(3%) to surgery wards. One hundred and twenty-seven (58%) strains were isolated from females and 76(42%) from males. Patients' mean age was 54±12 years (range: 26–81 years).

**ESBLs and prevalence ESBL-encoding genes**: Of the 203 isolates, 200(98.5%) showed reduced susceptibility to one of the five cephalosporins used in the primary screening. By performing confirmatory test, 127(62.6%) isolates were positive for ESBL production. Twenty-six (20.5%) isolates harbored the *bla*_OXA-1_ gene which was the most frequent gene, followed by 25(20%), 20(15.7%), 10(7.9%) and 2(1.6%) isolates carrying the *bla*_TEM-1_, *bla*_GES-1_, *bla*_CTX-M-15_, and *bla*_PER-1_ genes, respectively. The studied isolates were negative for the presence of *bla*_VEB-1_ and *bla*_SHV-1_ genes. Also, simultaneous presence of *bla*_OXA-1_-*bla*_GES-1_ genes was seen in 17(13.4%) isolates, *bla*_TEM-1_-*bla*_OXA-1_ genes in 7(5.5%) isolates, and *bla*_OXA_-*bla*_TEM-1_ genes in 4 (3.1%) isolates (Table 2).

**Table 2:** Repetitive-sequence-based PCR (rep-PCR) results of the ESBL-positive *A. baumannii* isolates

	No. of isolates (%)			

Genes	Type A	Type B	Type C	Total
*bla*_GES-1_+*bla*_OXA-1_	15 (11.8)	1 (0.8)	1 (0.8)	17 (13.4)
*bla*_TEM-1_+*bla*_OXA-1_	6 (4.7)	1 (0.8)	-	7 (5.5)
*bla*_TEM-1_+*bla*_GES-1_+*bla*_OXA-1_	7 (5.5)	-	-	7 (5.5)
*bla*_GES-1_+*bla*_OXA-1_+*bla*_CTX-M_	5 (3.9)	1 (0.8)	-	6 (4.7)
*bla*_OXA-1_+*bla*_CTX-M_	4 (3.1)	-	-	4 (3.1)
*bla*_TEM-1_+*bla*_GES-1_+*bla*_OXA-1_+*bla*_CTX-M_	2 (1.6)	-	-	2 (1.6)
*bla*_TEM-1_+*bla*_OXA-1_+*bla*_PER-1_	1 (0.8)	1 (0.8)	-	2 (1.6)

**Figure 1:**
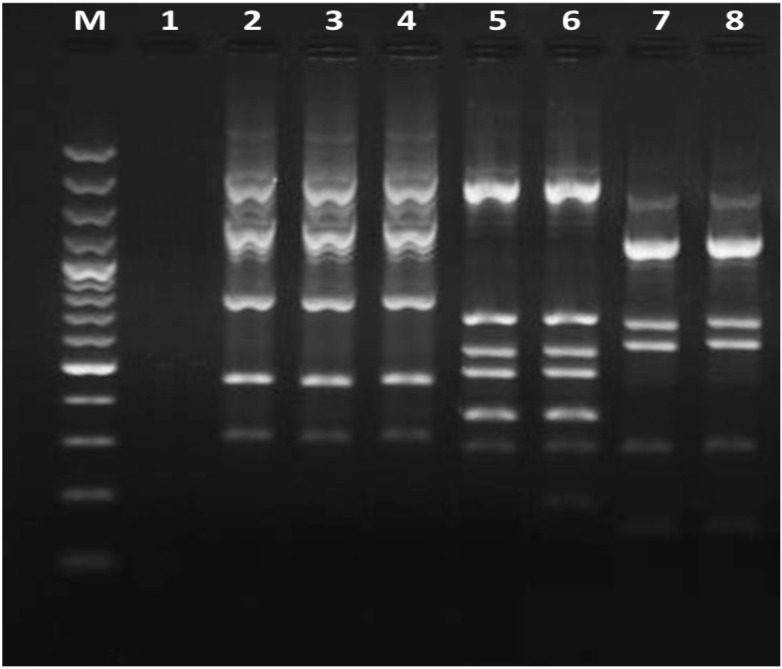
Representative REP-PCR fingerprints of Acinetobacter baumannii isolated from Qazvin hospitals. Lane M, 100 bp DNA ladder; lane 1, negative control (without template DNA), lanes 2 to 4, genotype A; lanes 5 and 6, genotype B; lanes 7 and 8, genotype C.

**Rep-PCR**: Rep-PCR results showed that ESBL-producing isolates belonged to 3 clones: A (85%), B (13.4%), and C (1.6%) indicating clonal spread of these resistant isolates among patients between different wards (Figure 1). As shown in Table 2, the presence of the *bla*_OXA-1_ gene alone or in combination was often shown in isolates belonging to clone A (50.3%) followed by clones B (5.6%) and C (0.8%).

## Discussion

Health care-associated infection caused by *A. baumannii* isolates is currently one of the major causes of patient mortality, especially in ICUs. Several reports from different geographical regions of Iran showed the presence of ESBLs in Gram negative bacteria (31–33). However, there was no study that investigated the presence of these enzymes in *A. baumannii* isolates in Qazvin province, central Iran.

In the current study, 62.6% of *A. baumannii* isolates were ESBL producers. Our results were consistent with two other studies in Iran in 2013 in Tabriz and in 2017 in Kermanshah on *A. baumannii* isolates which showed that 70% and 53.8% of the isolates produced ESBL, respectively (32,34). However, we found a higher prevalence of ESBL-producing *A. baumannii* compared with other studies in Iran in Hamedan (7%) and Tehran (16%) (35,36). The present study showed that ESBL-producing isolates were seen more in patients admitted to ICUs, internal and infectious wards and in trachea and urine specimens. Prolonged hospitalization and critical conditions for patients admitted to ICUs, the use of invasive procedures and devices, and prolonged exposure to broad-spectrum antibiotics may facilitate infection with these resistant organisms.

It seems that the presence of these resistant organisms in the studied hospitals may be due to inadequate management of infection control and lack of proper and rational medication administration. Our data emphasize the need to establish an antimicrobial resistance monitoring system locally and nationally and can effectively monitor the possible occurrence of this resistant organism in our healthcare system. However, it should be noted that the distribution of these resistant hospitalized organisms varies in different regions of the world. Factors such as age, severity of infection, patient immune response, method of administering broad-spectrum antibiotics, length of hospital stay and aggressive diagnostic and treatment methods considered for the patient may affect the incidence and prevalence of these infections.

In the present study, 20.5%, 20%, 15.7%, 7.9%, and 1.6% of isolates carried the *bla*_OXA-1_, *bla*_TEM-1_, *bla*_GES-1_, *bla*_CTX-M-15_ and *bla*_PER-1_ genes, respectively. In one study in Iran, the prevalence of *bla*_TEM_ and *bla*_CTX_ genes was 52.1% and 43.4%, respectively, in ESBL-producing *A. baumannii* isolates (36). Two other studies also reported the presence of *bla*_CTX-M_ and *bla*_TEM_ genes in clinical isolated of ESBL-producing *A. baumannii* isolates (34,37). In 2019, 42% of *A. baumannii* strains isolated from patients admitted to hospitals in Tehran were positive for the *bla*_TEM_ gene and negative for the presence of the *bla*_SHV_ gene (33). Rezaee et al. in 2013 found that 37% of the *A. baumannii* isolates harbored at least one of the *bla*_TEM-1_ and *bla*_PER-1_ genes, and none carried the *bla*_SHV_ gene (38). We previously showed the presence of *bla*_OXA-1_, *bla*_GES-1_, and *bla*_VEB-1_ in clinical isolates of *Pseudomonas aeruginosa* (39). In Iraq, Ghaima and colleagues found that 75% and 45% of *A. baumannii* isolates carried *bla*_TEM_ and *bla*_CTX-M_ genes, respectively (40). In another study, the prevalence of *bla*_TEM_ and *bla*_SHV_ genes was 8.8% and 25%, respectively (41). In Turkey, the frequencies of TEM, SHV and GES in ESBL-producing *A. baumannii* isolates were 55.7%, 7.7% and 1.5%, respectively (42). Taken together, these results confirm the presence of different ESBLs-encoding genes in *A. baumannii* isolated from medical centers worldwide. Several recent studies indicate an increasing presence of less prevalent ESBLs genes (*bla*_GES_, *bla*_PER_, and *bla*_SHV_) in *A. baumannii* isolates (38,43).

In this study, rep-PCR results showed that ESBL-producing *A. baumannii* isolates belonged to three separate clones which was strongly influenced by the clonal spread of these resistant isolate as well as patient-to-patient transmission. Moreover, we found that the dissemination of ESBL-producing isolates in different parts of the hospital as well as the dissemination of ESBLs-encoding genes occur in a clonal manner. This issue necessitates the use of appropriate infection control tools, especially in ICUs.

Our findings showed the significant presence of ESBL-producing *A. baumannii* isolates in the studied medical centers. This is the first report of the presence of *bla*_OXA-1_ gene in these isolates in Iran. Since the ESBL-producing isolates are often resistant to other antibiotics, they have created serious limitations and difficulties in the treatment of infected patients. Given the plasmid nature and rapid and widespread dissemination of ESBLs in treatment centers, early identification of these resistant organisms, the use of comprehensive antimicrobial treatment guidelines based on laboratory data and the use of appropriate infection control tools to prevent further infection are essential.
